# Impact Localisation in Composite Plates of Different Stiffness Impactors under Simulated Environmental and Operational Conditions

**DOI:** 10.3390/s19173659

**Published:** 2019-08-22

**Authors:** Aldyandra Hami Seno, M.H. Ferri Aliabadi

**Affiliations:** Department of Aeronautics, Imperial College London, Exhibition Road, South Kensington, London SW7 2AZ, UK

**Keywords:** composite materials, impact detection, impactor stiffness, environmental conditions, operational conditions, structural health monitoring, passive sensing

## Abstract

A parametric investigation of the effect of impactor stiffness as well as environmental and operational conditions on impact contact behaviour and the subsequently generated lamb waves in composite structures is presented. It is shown that differing impactor stiffness generates the most significant changes in contact area and lamb wave characteristics (waveform, frequency, and amplitude). A novel impact localisation method was developed based on the above observations that allows for variations due to differences in impactor stiffness based on modifications of the reference database method and the Akaike Information Criterion (AIC) time of arrival (ToA) picker. The proposed method was compared against a benchmark method based on artificial neural networks (ANNS) and the normalised smoothed envelope threshold (NSET) ToA extraction method. The results indicate that the proposed method had comparable accuracy to the benchmark method for hard impacts under various environmental and operational conditions when trained only using a single hard impact case. However, when tested with soft impacts, the benchmark method had very low accuracy, whilst the proposed method was able to maintain its accuracy at an acceptable level. Thus, the proposed method is capable of detecting the location of impacts of varying stiffness under various environmental and operational conditions using data from only a single impact case, which brings it closer to the application of data driven impact detection systems in real life structures.

## 1. Introduction

During its lifetime, an aircraft is subjected to multiple impact events from various sources (such as tool drop, bird strikes, debris, hail, etc.) under various scenarios (impact angle, mass, energy, etc.) and conditions (temperature, vibration, etc.) [1,2]. Unlike in metallic structures, where impacts generate visual dents [3], in composite structures, these impacts may produce barely visual impact damage (BVID), which may significantly reduce the residual strength of a structure and may result in catastrophic failure if left undetected [3,4]. As the name implies, BVID is difficult to detect using simple methods such as visual inspection [3]. Therefore, there is interest in development of systems to monitor the location and the severity of impact events to alleviate the need for frequent inspection using complex methods [5,6,7].

Impacts generate lamb waves that propagate outwards from the impact location as the source [8,9,10]. These waves have been used to identify the location of impacts using multiple methods available [8,9,10,11,12,13,14,15,16,17]. Methods that are based on the physical behaviour of wave propagation in the observed structure may require complex modelling/solving [8,15,18] or may not be suited to complex structures (cut outs, etc.) where wave propagation is indirect [9,19,20]. Data driven methods are able to generate meta-models that directly map lamb wave features to a specific impact coordinate, thus bypassing the need for physical models and giving flexibility to accommodate complex structures [11,12,21,22]. However, data driven methods are only accurate for the scope of initial data used for reference for training or as database [11,23,24].

Most of these methods were developed for impact localisation in ideal/lab conditions, which does not represent the variation caused by different impact cases (scenarios, conditions, and sources) in real life [1,2]. These variations may alter the generated lamb wave, increasing the complexity of physical models required or, in the case of data driven methods, may alter the response to differ from the original reference data, which dramatically reduces their accuracy [23,24]. Although incorporating variations in the reference data of data driven methods may mitigate the accuracy loss [11,12], it is not feasible in real life, as the possible combination of parameters is very large.

In a previous study [23], it was found that minute variations (amplitude, waveform, etc.) in the generated lamb waves between different impact cases (energy, mass, angle, temperature) may cause variation in extracted features depending on the extraction methods used, not necessarily because of actual change in the lamb wave propagation characteristics. A feature extraction method was then developed for artificial neural network (ANN) based impact localisation that was able to extract consistent features (or eliminate variations) for impacts of different cases whilst filtering out operational vibration noise. This meant that by using data from a single impact case, we were able to accurately locate impacts under various combinations of the mentioned parameters, leading to a significantly smaller initial data set required and allowing feasible localisation of real life impact cases. 

However, besides the parameters assessed in the study [23], variation of impacts can also come from differences in impactor stiffness caused by different impactors (debris, bird strike, tools, etc.) [10]. Out of the few studies that have investigated the effect of impactor stiffness on the generated lamb waves, it was found that lower impactor stiffness generates significantly lower frequency lab waves [10,15,18,25]. These differences can significantly affect feature extraction [10,18], which in turn affects the accuracy of data driven localisation, as the difference in stiffness of a test case compared to the initial training case becomes larger [25].

Thus, in order to improve a previous study [23], here we develop a novel data driven impact localisation methodology that is not only robust for previously studied cases (velocity, mass, angle, temperature, vibration noise) but also robust for detecting impacts of different stiffness whilst using an initial data set from only one impact case. In order to accomplish this, we conducted a novel parametric comparison of experimental data on impacts for the cases of interest. We then used the observation results to develop a methodology, which was tested against our previously developed method as benchmark [22].

## 2. Experimental Setup

**Figure 1:**
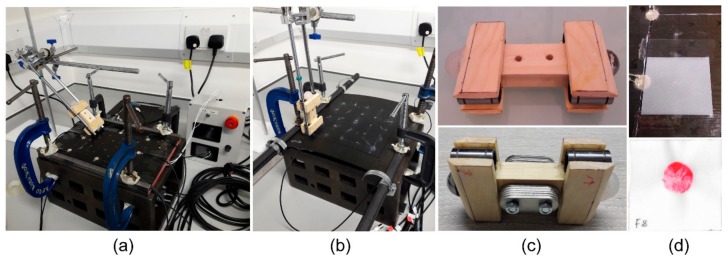
Impact setup: impact tower (**a,b**), flat plate (**a**), curved plate (**b**), impactor (**c**, **top**) with 2 heads (steel and silicone) and added mass (**c**, **bottom**), paper for contact area measurement (**d**, **top**) and example scan from soft impact (**d**, **bottom**).

Experimental impact data under different scenarios (impactor stiffness, angle, mass, energy) and conditions (temperature, vibration) were gathered using a multiparametric impact setup designed in a previous study [23]. The setup consisted of a drop impact tower, a fixture for holding specimens, two composite plate specimens (flat and curved) with piezoelectric (PZT) sensors and an oscilloscope to measure the voltage from the PZT sensors, as shown in Figure 1.

**Figure 2:**
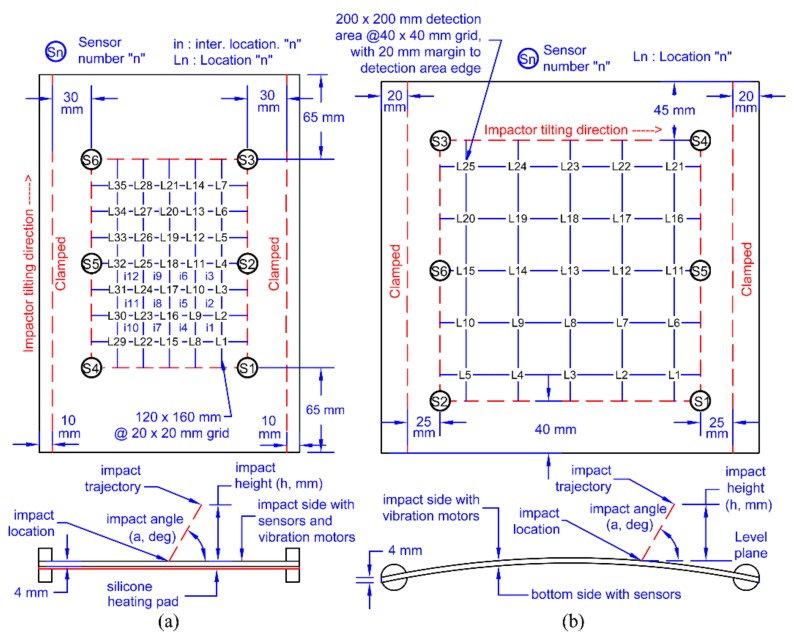
Layout of flat (**a**) and curved (**b**) composite plates.

The impact fixture (Figure 1a,b) was made up of steel bars, which held the plates via clamps on to a base. Both plates were made of M21 T800s carbon fibre prepregs with a 16 layer quasi-isotropic lay up ([0/+45/-45/90/0/+45/-45/90]_s_) resulting in a thickness of 4 mm. The flat plate was 200 × 290 mm with eight PZT sensors bonded (but only six were used in this study to match the curved plate) to the top side of the plate (impact side) and had a silicone heating pad on the bottom side for temperature control (Figure 1a). The curved plate was 290 × 285 mm with six PZT sensors bonded to the underside of the plate (opposite to the impact side) to mimic the conditions of an aircraft panel (Figure 1b). The layout of the sensors and the impact locations of both plates can be seen in Figure 2.

The impact tower consisted of the tower itself and the impactor (Figure 1). The impactor (Figure 1c) had an original mass of 100 gr and could be increased to 200 gr with added weights. There were two impact heads (both spherical, 20 mm diameter) with differing materials (silicone and steel) to simulate impacts from different stiffness materials. Impacts were generated by lifting the impactor to a certain height (controlling the energy) and then letting the impactor drop onto the plate. The rails of the impact tower guided the trajectory, thus allowing for angled impacts (Figure 1a and Figure 2). To measure the contact area during impact, the impactor heads were painted, and a 40 × 40 mm piece of paper was placed on the impact surface (held in place lightly with two strips of adhesive tape) to capture the print of the contact area (Figure 1d top). Each print was then scanned (Figure 1d bottom), and the contact area silhouette was extracted for further comparison.

Lamb waves caused by impact were transformed into voltage signals by the PZT sensors, which were recorded using an National Instruments (Austin, Texas, USA) PXI 5105 8 channel oscilloscope. The sensors were connected to the oscilloscope via probes, which were also used to attenuate (10x) the signals from hard impacts (impacts with the steel head), as the voltage magnitudes were too high for the oscilloscope. No attenuation was used for soft impacts (impacts with the silicone head). Signals from each of the six sensors were recorded at 2 MS/s sampling rate with a length of 100,000 samples using National Instruments (Austin, Texas, USA) SignalExpress software. All data processing and localisation algorithms were done using MathWorks MATLAB.

Table 1 and Table 2 give the list of impact case data sets recorded for the flat and the curved plates under various impact scenarios and conditions. For each plate, a reference impact case (F1 and C1) was used as the baseline, whereas all other impact cases were a deviation (change of one or two parameters) from the said reference. These reference cases also served as the training data sets for the localisation algorithms later on. 

Unlike in the previous study [23] when this setup was first used, vibration noise was not generated physically but rather added artificially in MATLAB, as this allowed for simulation of more severe noise magnitude (3V for hard impacts and 0.1V for soft impacts) and with a controlled random spectrum (as stated in RCTA/DCO-160C [26]).

## 3. Impact Signal and Contact Behaviour from Hard and Soft Impacts

### 3.1. Effect of Impact Case Variation on Impact Signal and Contact Behaviour

**Figure 3:**
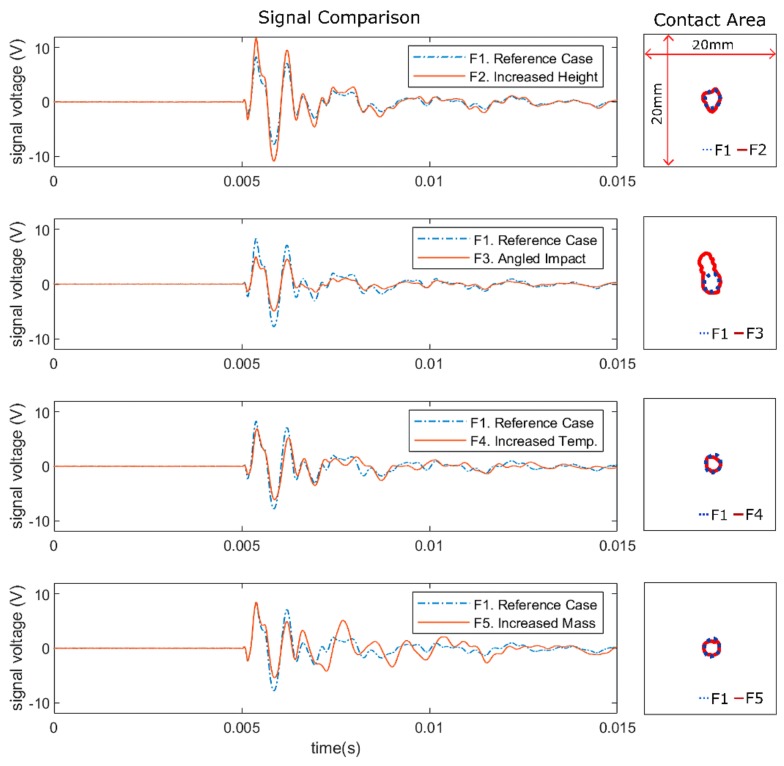
Comparison of impact signals (from sensor 6) and contact area from impacts at location 15 of the flat plate for hard impact cases (F1–F5).

**Figure 4:**
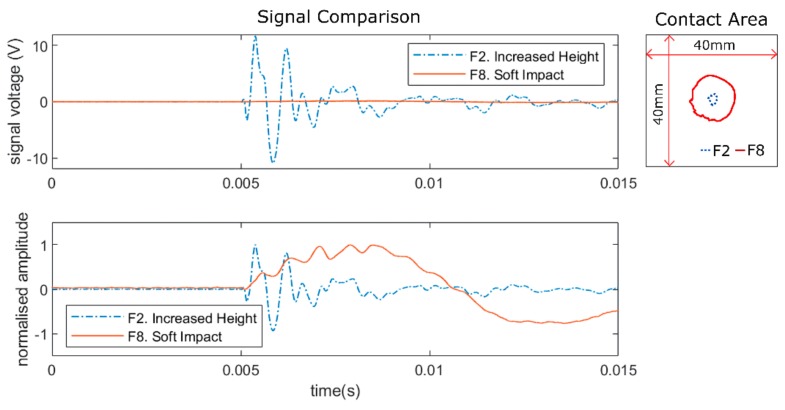
Comparison of impact signals (from sensor 6) and contact area from impacts at location 15 of the flat plate for cases F2 and F8.

As mentioned in Section 1, minute variation in lamb wave amplitude and waveform due to differing impact cases was found to significantly affect lamb wave feature extraction for impact localisation [23]. To compare the effects of variation in impact case on the contact behaviour and the subsequent lamb waves, we chose one impact location from the flat plate (as it had the complete set of parameter variations) to act as a reference point Figure 3 shows the comparison of recorded impact signals and measured contact area between the reference case (F1) and the cases F2–F5 at impact location 15 measured from sensor 6. As the soft impacts (F8) were conducted with a higher impact height than the reference case (due to the very weak signal produced by the soft impact), the comparison was conducted with case F2, as the heights (and thus energy) were the same (Figure 4).

Comparison between signals from case F1 and F2 showed that an increase in impact height (and subsequently velocity and energy) only affected the amplitude of the signal but not the waveform [23]. Comparison of the contact area revealed that, although the impact energy was higher, the contact area was mostly unchanged.

Since the vertical impact height for cases F1 and F3 were the same (25 mm), the initial potential energy and the resulting velocity (and thus kinetic energy) of the impactor at contact was also the same. However, for the angled impacts (F3), the transverse component of velocity was smaller compared to perpendicular impacts (F1) and thus resulted in a less severe impact, as can be seen by the smaller amplitude of the impact signal [4,23]. Observation of the contact area of the angled impact (F3) indicated a similar initial contact area proceeded by sliding contact due to the velocity components parallel to the contact surface, possibly generating the differences observed in the impact signal waveform. However, as both velocity components (transverse and parallel) were equal due to the 45° impact angle, it seems that the transverse contact was still the dominant source of the lamb wave, as the change in waveform was relatively small (possibly due to low friction coefficient between impactor and contact surface).

Comparison of impact signals between case F1 and F4 showed that increasing temperature induced as a slight shift in waveform due to changes in wave propagation velocity [27] and reduction in amplitude due to reduction in adhesive stiffness [28]. Observation of the contact area showed that the variation in temperature did not affect the contact area significantly.

Cases F1 and F5 both had the same impact height, thus the resulting impact velocity was the same (due to constant gravitational acceleration), which seemed to be mirrored in the first portion (up to the first positive peak) of both signals. However, as the impactor had larger mass in case F5, the momentum of the impactor was larger, and thus the momentum transfer differed from the reference case (F1), as shown by the significant difference in waveform of both signals after the first positive peak. Similar to the previous cases, the contact area was not significantly affected, even though the impact energy was larger. Thus, it could be concluded that, for hard impacts, the deformation of the impactor head was small and was not significantly affected by variations in impact case. 

Figure 4 shows the comparison between two identical impacts but with different stiffness impact heads (F2: steel, F8: silicone). Observation of the contact area revealed that the soft impact (F8) had a significantly larger contact area than the hard impact (F2) due to larger deformation of the impact head. This led to significantly smaller impact signal amplitude (Figure 4), as the kinetic energy was partially absorbed by the soft impactor head rather than transferred to the plate. This possibly also acted as a dampener for the impact signal, as the impact energy was transferred gradually when the impactor deformed, leading to a much lower frequency impact signal (as can be seen from the normalised signals in Figure 4). Contrast this to the hard impact, where the deformation was small and thus the energy transfer occurred faster, resulting in a high frequency signal. This difference in contact behaviour resulted in differences in impact signal amplitude and waveform that were far greater than any other impact case variation previously observed (cases F1–F5) and could create difficulties in consistent feature extraction, which is necessary for accurate localisation [18,23,25].

### 3.2. Simulation of Random Vibration Noise and Noise Filtering

**Figure 5:**
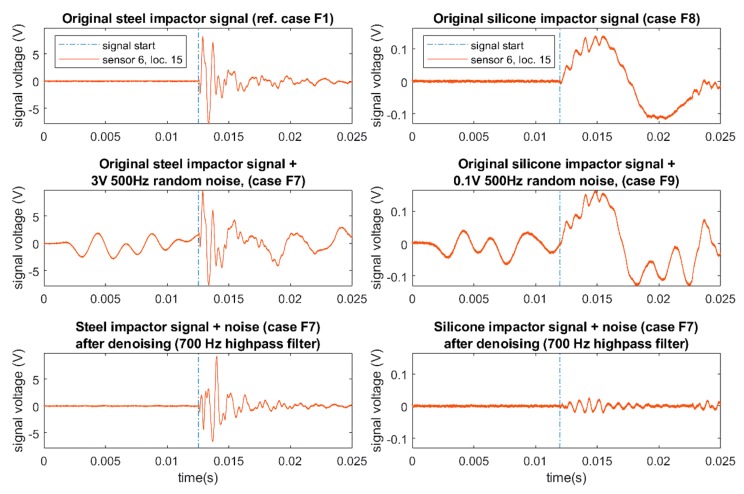
Comparison between hard (F1) and soft (F8) impacts at location 15 measured from sensor 6 of the flat plate: original (top), with random noise (middle), and after noise filtering (bottom).

As mentioned in Section 2, artificial random noise was added to the impact signals to simulate vibration noise during operation. This noise was generated for each impact signal using MATLAB, where a series of random numbers was generated (creating white noise with bandwidth up to 1 MHz or half the sampling frequency) with the same length as the signal (100,000 samples), after which a 500 Hz low pass Butterworth filter was applied to limit the bandwidth of the random noise (although some small components just after the 500 Hz cut off frequency, around 700 Hz, still existed, as the filter had not reached maximum attenuation). This bandwidth was chosen from the frequency limit of the soft impacts, as is demonstrated later on. The amplitude was then scaled to 3 V for the hard impacts and 0.1 V for the soft impacts (due to the much smaller amplitude of soft impacts collected) to give a moderate magnitude of noise. This noise was then superimposed to the impact signals of the reference cases (F1 and C1) and also the soft impacts (F8 and C5) from both the flat and the curved plates (creating impact cases F7, F9, C4, C6). Figure 5 shows the noise added to the signals measured from sensor 6 of the reference case (F1) and the soft impact (F8) at location 15 of the flat plate. It can be seen that the noise masked the signal, especially the start, which is vital for feature extraction such as time of arrival (ToA) for localisation purposes.

As impacts generate signals with a wide bandwidth [23,29,30], high pass filtering is commonly used to remove vibration noise, as it usually has a lower bandwidth limit [5,23,26]. Here, we applied a 700 Hz Butterworth high pass filter to completely remove all components of the previously added vibration noise. As can be seen in Figure 5, for the hard impact after high pass filtering, there was still a significant portion of the impact signal left to be able to identify the start of the signal. However, as soft impacts generated impact signals with lower frequency (narrower bandwidth), there was little left of the signal after high pass filtering, making it more challenging to pick the start of the signal accurately. Thus, the frequency bandwidth of soft impacts (near 700 Hz for this data set) limited how much noise filtering could be done before the impact signal itself was completely lost and the impact became undetectable using this method.

## 4. Feature Extraction from Hard and Soft Impacts for Localisation

For localisation algorithms, especially data driven methods, the main interest is to lower the amount of initial data required for training or building a reference database, as collecting large amounts of data (from the large combination of different impact scenarios and conditions) is not practical for large and complex structures such as aircrafts [13,23,25,31]. To obtain accurate results by using an initial data set from only one impact case, the features extracted from impacts under different cases must be as consistent as possible to match that of the initial data set [23]. 

From previous studies, it has been shown that wave ToA difference (as the actual time of impact is unknown, thus it is in terms of difference towards the first arriving signal) is the best feature for localisation [12], followed by signal amplitude ratios [12,32]. Thus, in this section, we look into extraction of these two features. In our previous study [23], a ToA extraction method was developed that could homogenize the waveform and the amplitude variations (Section 3.1) and obtain consistent ToA for hard impacts under various impact cases. However, as shown in Section 3, soft impacts generate impact signals that are significantly different than hard impacts and thus may have significant effects on consistent feature extraction. Thus, in this section, we observe the effects of differing impactor stiffness on feature extraction and develop methods to obtain consistent results.

### 4.1. Normalised Smooth Envelope Threshold (NSET) Method for ToA Extraction

The normalised smooth envelope threshold (NSET) algorithm was developed to extract consistent ToA from hard impact under various impact cases (differing energy, mass, angle, and temperature) [23]. The algorithm first converts the signals into absolute values, and then an envelope is constructed by running the absolute valued signals through a low pass filter. This removes all the small peaks at the start of the signal and creates a smooth initial rising edge. Afterwards, the amplitude of each signal envelope is normalised by the largest amplitude of the envelopes from all sensors. The ToA value is then determined from the envelope using thresholding (the signal is said to arrive when the amplitude surpasses a certain threshold). The extracted ToA is more consistent, as it is less sensitive to the threshold value due to the smoothing done by the low pass filter.

**Figure 6:**
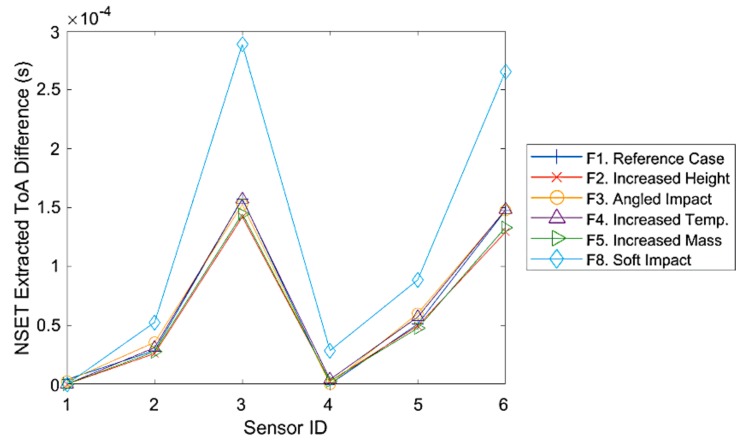
Comparison between time of arrival (ToA) difference extracted from hard (F1–F5) and soft (F8) impacts at location 15 of the flat plate using the normalised smoothed envelope threshold (NSET) method (2 kHz low pass filter, 2.5% of maximum amplitude threshold).

It was found that this method coupled with high pass filtering to remove vibration noise was successful in extracting consistent ToA for hard impacts under various impact cases (F1–F5), as can be seen in Figure 6. However, when tested on soft impacts (F8), it was found that, although the ToA profile/pattern was similar to hard impacts, the ToA value showed significant variation between hard and soft impacts (Figure 6). This was most likely due to the greater difference in impact signal waveform caused by differing impact stiffness (as observed in Section 3.1) compared to any other parameter variance (energy, temperature, mass, and angle), which could not be completely homogenized by the normalisation and the low pass filter envelope used in the NSET method.

This difference in ToA caused large localisation errors for soft impacts (F8) when using ANNs, as the features were not consistent with the reference case (F1), which was used to train the localisation algorithm [23,24]. Thus, either a more consistent ToA extraction method or a less sensitive localisation algorithm was needed (in this paper, we chose the latter, as detailed in Section 5).

Additionally, since this method relies on a threshold value to determine the start of the signal, it is not suitable for very small signals with low signal to noise ratio (SNR), as in these cases, the threshold has to be very low to detect signals and then has high risk of false detection due to noise. As shown previously in Figure 5, the signals left behind after high pass filtering soft impacts were very small and thus not feasible to detect using this method. Thus, a new ToA extraction method was required that was more robust in extracting the signal ToA of very small signals. 

For the purpose of validating new methodologies, the NSET method was used as a benchmark. A Butterworth 2 kHz low pass filter was used for smoothing, and a threshold was set at 2.5% of the maximum amplitude of the signals in the array.

### 4.2. Modified Akaike Information Criterion (AIC) Method for ToA Extraction

A signal can be divided into a number of locally stationary processes that can be stochastically approximated using autoregressive models [9,33]. In the case of an impact signal, it can be divided into two segments—the period before the impact signal arrives, which is noise, and the period after the impact signal arrives, which is dominated by the impact signal itself (as can be seen in Figure 3, Figure 4 and Figure 5). As the two segments are stochastically different, the best possible fit of the autoregressive models is achieved when the selected separation point between the two segments coincides with the arrival time (ToA) of the signal. Thus, using this criterion, we could determine the ToA of a signal by finding the separation point that produced the best fit for the autoregressive models.

The Akaike Information Criterion (AIC) gives the relative quality of fit between different statistical models for a given data set [9,33]. By calculating the AIC for differing separation points along the length of the signal, we could find the point where the AIC value was minimum, which indicated best fit of the autoregressive models and subsequently the ToA. Thus, unlike other ToA pickers [23,25,34,35], the determination of ToA was not dependent on a predefined magnitude threshold, making it more feasible for ToA picking of small signals with low SNR [36]. 

Equation (1) shows how the AIC value was calculated for each possible separation point (*t*) along a signal (*x*) with a certain length “L” as derived by Maeda [33] and Simone [9]. For each step, the lengths of each segment (*x_1 ~ t_* and *x_t+1 ~ L_*) changed as the tested separation point (*t*) moved from the start of the signal to the end. The AIC value at each step was calculated to evaluate the goodness of fit for the given separation point (*t*).

(1) $$AIC\left(t\right)=t\ln\left(var\left(x_{1\;\sim \;t}\right)\right)+\left(L-t-1\right)\ln\left(var\left(x_{t+1\;\sim \;l}\right)\right)\;),\;t\;=\;1,2,\ldots L$$

**Figure 7:**
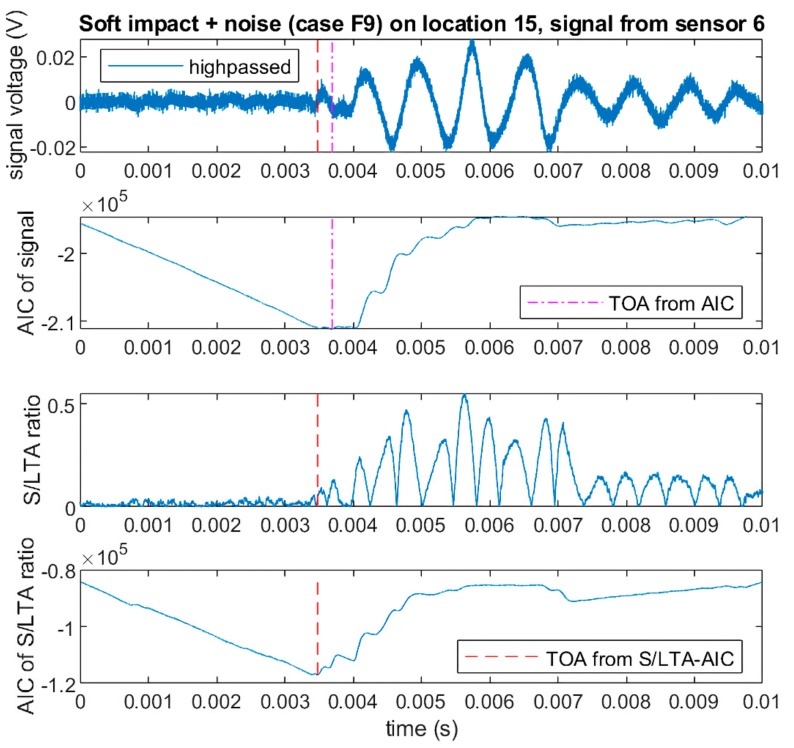
Comparison of ToA extracted using Akaike Information Criterion (AIC) and Short Term/Long Term Average ratio (S/L TA) AIC for high passed filtered signal from soft impact with noise (F9) measured by sensor 6 at location 15 of the flat plate.

Figure 7 shows the AIC values calculated for the high passed signal from the soft impact with added noise (F9) shown in Figure 5. To save computational time, we used a rough threshold above the noise level (4 V for hard impacts and 0.125 V for soft impacts) on the original (not filtered) signal to obtain a rough estimate of the region where the signal started. Then, a window of 10,000 sample points before and after the triggered point was taken as a short window for AIC calculation. It can be seen that the minimum value of the calculated AIC (thus the predicted ToA) was near the start of the signal. However, it can also be seen that the minimum of the AIC was not clearly pronounced and showed a “flat” region where the AIC values were similar and led to uncertainty in choosing the minimum point. This occurrence was most likely due to the very small amplitude of the signal, which was difficult to discern from noise.

Previous studies [9,35] developed characteristic functions (CF) to enhance certain features of the signal (amplitude, SNR, etc.) in order to gain more accurate ToA. Examples of CFs include signal absolute value, squared signal, signal gradient, and Hilbert transform [9,35]. However, for signals such as those shown in Figure 7 where the area of interest (the start of the signal) is much smaller in amplitude than the rest of the signal, CFs such as absolute value, signal gradient, and Hilbert transform [9,35] may not always significantly affect the said area of the signal. Moreover, squaring the signal may instead minimise the start of the signal (as it is small in amplitude) and amplify the larger peaks instead. 

Thus, we proposed a novel CF that could enhance the sudden changes caused by the arrival of the impact signal. The proposed CF was based on the Short Term/Long Term Average ratio (S/L TA) picker [34], where two averages with different sliding window lengths (short and long) are calculated for the length of the signal, and a ratio is calculated between them. The short term average reacts to sudden changes in the signal, whilst the long term average has a more stable value. Thus, when the short term average is divided by the long term average, sudden changes at the start of the signal (where the long term average is still low since it is all mostly still noise) are enhanced, whilst larger amplitudes later on (where the long term average is higher due to the signal) with less change are not. However, for the small signals, as in Figure 7, it was found that the calculation of the ratio was prone to instabilities due to the averages (especially the long term as the denominator) having very small values at some areas of the signal. Thus, before calculation, the original signal (*x*) was shifted by 1.5, the maximum absolute amplitude, resulting in a positive signal (*xp*) where no amplitude was near zero. Equation (2) shows how the CF (named the S/L TA ratio) was calculated for a positive signal (*xp*) of length “L” with short window length “ws” and long window length “wl” (in this study, we used 25 and 500 sample points for the short and the long window lengths).

(2) $$S/LTA\;ratio\left(t\right)=\;\frac{mean\left(xp_{t-ws\sim t}\right)}{mean\left(xp_{t-wl\sim t}\right)}\;-1,\;t\;=\;1,\;2,\ldots L\;,\;t>wl$$

**Figure 8:**
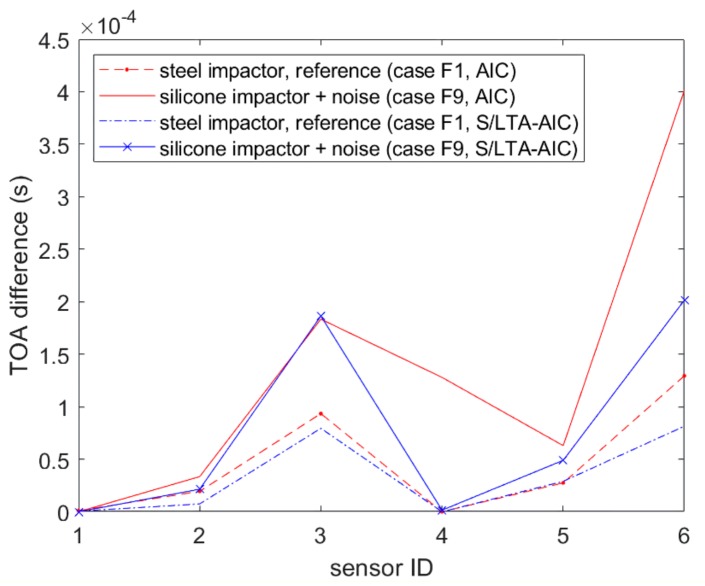
Comparison between ToA of the reference case (F1) and the soft impact with noise (F9) extracted using AIC and S/L TA-AIC method.

Figure 7 shows the S/L TA ratio calculated for the high passed soft impact signal shown in Figure 5, where the resulting signal had less noise and a more pronounced peak at the arrival of the impact signal. When the AIC was calculated for the S/L TA ratio instead of the original signal, it could be seen that the AIC minimum was much clearer, and the corresponding ToA was more accurate. From here on, we call this method the S/L TA-AIC method. Figure 8 shows the comparison of ToA profiles for all six sensors between soft (F9) and hard impacts (F1) obtained using the AIC and the S/L TA-AIC method for impacts at location 15 (Figure 2) of the flat plate. It can be seen that the S/L TA-AIC method gave more consistent ToA profiles between the soft and the hard impacts, which resulted in more accurate localisation of soft impacts. Thus, this method gave a robust way of determining the ToA from high pass filtered soft impact signals that had very low amplitude. However, significant variation between ToA of hard and soft impacts remained, which significantly affects localisation accuracy. 

### 4.3. Signal Amplitude

As shown in Section 4.1 and Section 4.2, soft impacts generated significant variation in ToA between hard and soft impacts, which may cause significant reduction in localisation accuracy. Thus, here, we looked at an alternative feature to be used for localisation of impacts with differing stiffness. Besides ToA, signal amplitude has been shown to be a good feature for localisation [12,32], as there is strong correlation between impact distance from a sensor and signal amplitude (closer impacts generate larger amplitude signals and vice versa) [32]. As the signal amplitude itself changes with differing impact cases (energy, mass, etc.), the amplitudes (we used the minimum amplitude here) are normalised such that the input is the ratio of impact signal amplitudes between sensors in an array. 

With respect to vibration noise, for severe impacts (which are of the main interest for localisation), the noise level should be much smaller than the amplitude of the signal and thus possibly negligible. Therefore, we still used the amplitudes from the original signal rather than the filtered signal, as the frequency bandwidths of different impact cases differed (especially soft and hard impacts), and the consistency of the amplitude ratios may have change when some components of the waves are filtered. 

## 5. Impact Localisation Methods

In Section 4, we discuss the developed feature extraction methods that can improve the feasibility and the consistency of feature extraction for hard and soft impacts. However, significant variation of extracted features still exists, which may significantly reduce localisation accuracy using data driven methods [23,24]. Thus, we developed a localisation method that was less sensitive to input feature variations.

### 5.1. Artificial Neural Networks (ANNs)

ANNs are a network of mathematical operations between an input and an output that are linked together via weighted connections [23,29,37]. ANNs can create a meta-model mapping given input and output via training using a previously known input and output data set. During training, the ANN is given the input from the training data, and the weights of the connections are optimized to fit the output of the ANN to the output of the training data. 

From previous studies [11,18,23,24,25,29,32,38], it was found that ANNs are able to accurately locate the position of an impact using input in the form of impact signal features such as ToA and signal amplitude. However, the accuracy is significantly reduced when there is variation between input features from an impact at a given location that differs from the features for the said location in the original training set. As the mapping of the ANN is only calibrated to what is included in the training set, when given an input that is outside of what is contained in the training set, the ANN may not be able to map it accurately [11,18,23,24,25,38]. Thus, to ensure the accuracy of localisation, either a training set containing all possible variations of the signal features or a method to ensure consistency of extracted features is needed. This is a significant problem for real life application, as there are multiple parameters that may alter the recorded impact signal and subsequently the extracted features (as shown in Section 3) [18,23,24]. The possible combination of these parameters is very large and would require collecting an impractically large training set. 

As mentioned in Section 4, we previously [23] developed a method to extract ToA consistently for hard impacts under various impact scenarios and conditions, which allows for accurate localisation of these impacts using ANNs with a training set consisting of only a single impact case. However, for soft impacts, we had yet to be able to reach the same consistency, and thus we required an alternative localisation method. 

For validation purposes of new localisation methods, we used ANNs as benchmark for comparison. The ANNs had a single hidden layer with 12 neurons with ToA as input and the predicted impact coordinates (x,y) as output. To account for random initial weights, each ANN training (done using the Levenberg–Marquardt algorithm) was repeated 500 times, and the best performing iteration was chosen as the ANN to use [23]. 

### 5.2. Reference Database Method (DTB Method)

The reference database (DTB) method [13,14,16,39] is similar to ANNs, as it also utilizes an initial data set/database of previously known inputs and outputs to predict the location of an impact. Features of an incoming impact wave are compared to the known features corresponding to different impact locations stored in a reference database. The reference location, which has corresponding features that have the least difference compared to the features of the incoming wave, is considered to be the location where the impact most likely occurred. As the determination of impact location is based only on similarity, this method is less sensitive to variation in input features and should be able to give acceptable location prediction provided there is some degree of similarity between the input features and the features stored in the database. In Section 4, we show there is a degree of similarity between the features extracted from hard and soft impacts with variation occurring to differing degrees on each sensor. Thus, we used the reference database method as an alternative to ANNs for localisation of hard and soft impacts. 

The difference (*D_i_*) between the input features (*y^in^*_j_) and the reference features (*y^ref^_i,j_*) from “Ns” number of sensors stored in a database containing “Nr” number of reference features is given in Equation (3). The value of D_i_ was calculated for all sets of reference features stored in the database, where the location coordinates (*x_i_, y_i_*) corresponding to the set of reference features that minimized D_i_ were said to be the impact location.

(3) $$D_{i}=\sum_{j=1}^{Ns}\;y_{j}^{in}-y_{j,i}^{ref},\;i\;=\;1,2,\ldots Nr,\;j\;=\;1,2,\ldots Ns$$

As noted in Section 4, the degree of variance between hard and soft impact features differs between sensors. Thus, we proposed a novel comparison method where, rather than determining the location using all the sensors at once (*j* = 1,2,…*Ns*), we split the sensors into groups of combinations of “Ncs” number of sensors (for example, Ns = 1–6, *Ncs* = 5, *j*_1_ = 1,2,3,4,5, *j*_2_ = 1,2,3,4,6, *j*_3_ = 2,3,4,5,6, etc.) and produced multiple impact location predictions (*x*_1_, *y*_1_; *x*_2_, *y*_2_; *x*_3_, *y*_3_; etc.). These predictions were then averaged to obtain the final impact location prediction (*x*,*y*). The idea was that, by using different combinations, the probability of having a combination of exactly all sensors with large feature variation was smaller than the probability of having a combination where one or more of the sensors with large variation was not included. Thus, out of all the location predictions produced, most were likely to have originated from combinations with less feature variation and subsequently had better accuracy.

**Figure 9:**
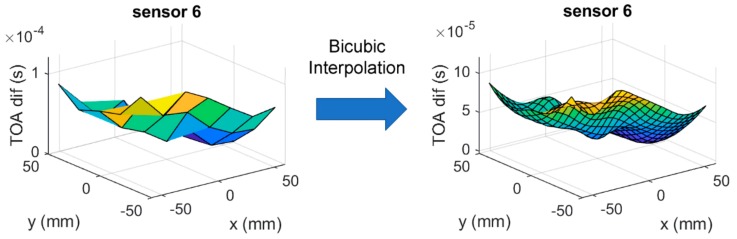
Bicubic interpolation of ToA extracted using the S/L TA-AIC method from sensor 6 of the flat plate for the reference case (F1).

As the reference database consists of discreet locations and their corresponding features, to predict impact locations over a continuous area, there needs to be an interpolation method between the reference locations. This is commonly done by either interpolating the impact location from a set of reference locations with the highest similarity to the input features [16,39] or by interpolating the reference database entries to gain a smoother discretisation (thus approximating continuity) of the sensing area [14]. Here, we chose to interpolate the database entries due to the simplicity, where the grid shown in Figure 2 was discretised by the order of two using bicubic interpolation (Figure 9). 

## 6. Impact Localisation Results

In this section, we conduct localisation of the impacts collected in Section 2 (Table 1 and Table 2) using the methodologies developed in Section 4 and Section 5 based on observations done in Section 3. In order to validate these methods, we conducted comparisons of these methodologies with a benchmark method which, in this case, was the localisation method using ANNs with ToA extracted using the NSET method (Section 4.1 and Section 5.1). The main objective was to see if the proposed methodologies could improve localisation of impacts with different stiffness using training/reference data only from impacts with a single stiffness compared to the benchmark method.

The data set used for the ANN training set and the reference database in this section was taken from the reference impact cases (F1 and C1) where one out of four repeated impacts at each location (35 and 25 impacts for the flat and the curved plates, respectively) were collected into a training/reference data set. The extracted features from these impacts were then interpolated (as mentioned in Section 5.2) to produce a fine discretisation for the reference database and at the same time produce a large enough database for training the ANN. The rest of the impacts from the reference cases (105 and 75 impacts for the flat and the curved plates, respectively) were used as testing data for cases F1 and C1.

Impact localisation accuracy was quantified using the Root Square Error (RSE) of the predicted impact location towards the actual impact location [12,23,24]. To express the general localisation accuracy of a whole data set, we fit a gamma distribution (as there were cases of zero error, the commonly used lognormal distribution could not be fit) [12,23,24] to the RSE of all predicted impact locations in the data set and took the RSE value corresponding to 90% on the cumulative distribution function (CDF) of the fitted distribution. We defined this RSE value as the range around the predicted impact location where we had 90% confidence that the actual impact location was located within (smaller value is better) or 90% Probability of Detection (POD) [12,23,24].

### 6.1. Comparison between Localisation Methods

In this section, we compare the accuracy of the benchmark localisation method with the proposed reference database method with varying amounts of sensor combinations when tested with impacts with different stiffness (soft impacts, F8 and C5) from the training/reference database (Table 3). The input used was ToA extracted using the NSET for all cases to isolate the localisation accuracy improvements due to the localisation method alone. 

**Figure 10:**
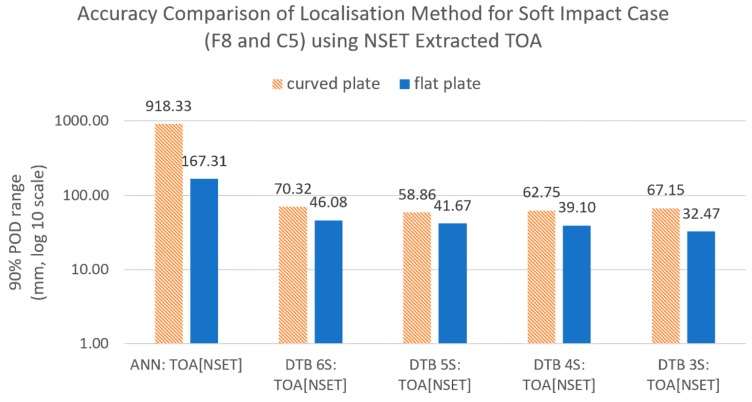
Comparison of localisation accuracy for different methods trained with the reference case (F1 and C1) and tested with the soft impact (C5 and F8).

**Figure 11:**
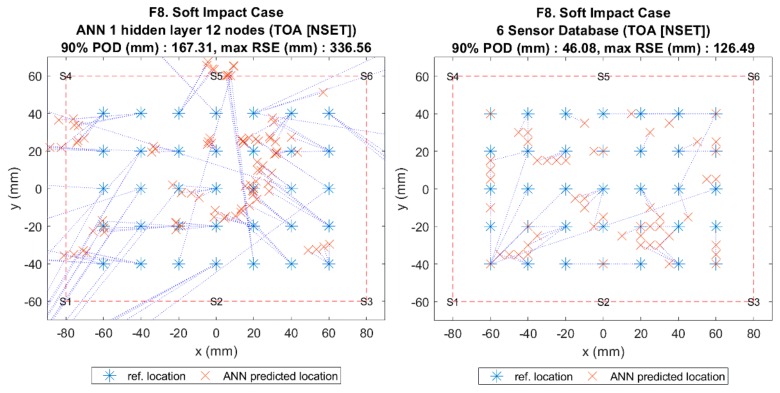
Visualisation of impact localisation for soft impacts (F8) using different methods: ANN (left) and reference database (right).

The results of the comparison can be seen in Figure 10 and Figure 11, which show that, when using the benchmark method (ANN with NSET ToA), the localisation error for soft impacts was very high. However, when the same input was given to the reference database method without sensor combinations (all six sensors), the error dropped significantly. This suggested that the ANN was very sensitive to the variations in ToA between the training case (F1 and C1) and the soft impact test case (F8 and C5), whereas the reference database was much less sensitive due to the mapping being based on similarity only. 

When tested using the reference database with varying degrees of sensor combinations, it can be seen that using sensor combinations decreased the error further. However, although it was found that the error decreased with increasing number of sensor combinations for the flat plate, for the curved plate, the largest decrease was achieved with five sensor combinations. This suggested an optimum number of sensor combinations dependent on the structure. For later comparisons, we used five sensor combinations for the reference database to ease comparison between the flat and the curved plates.

### 6.2. Comparison Between Input Features for Localisation

In this section, we compare the effect of different input features on soft impact (F8 and C5) localisation accuracy. To isolate the effect of various inputs, the localisation method used for all cases was the same (reference database method with five sensor combinations, Table 4). For the case of multiple input features, such as ToA with minimum amplitude ratio (Table 4), two separate reference databases were used for each input feature with the sensor combinations done separately for each database. The predicted coordinates of these separate reference databases were then averaged to obtain the final prediction. The localisation results using the benchmark method (ANN with NSET ToA) were also included as reference for comparison. 

**Figure 12:**
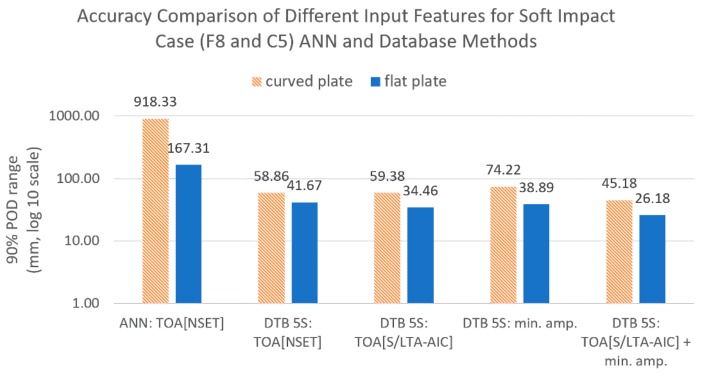
Comparison of localisation accuracy for different input features, all trained with features from the reference case (F1 and C1) and tested with the soft impact (C5 and F8).

**Figure 13:**
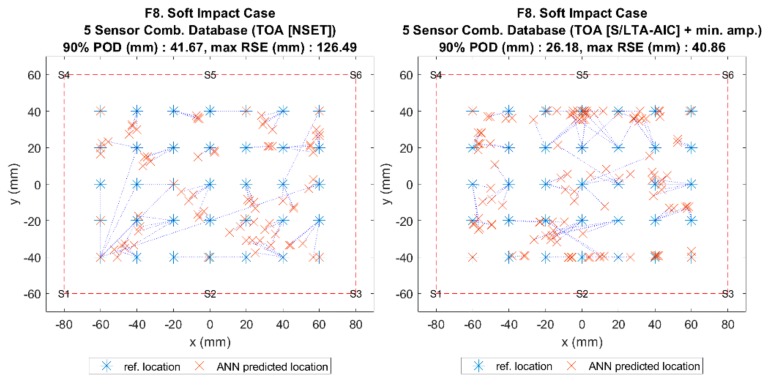
Visualisation of impact localisation for soft impacts (F8) using different input features NSET extracted ToA (**Left**) and S/LTA-AIC extracted ToA with normalised minimum amplitude (**right**).

Figure 12 and Figure 13 show the comparison results of localisation accuracy for various input features. Comparing the different ToA extraction methods, it can be seen that, for the flat plate, the S/L TA-AIC method gave lower localisation error than the benchmark NSET method. However, the same was not observed in the curved plate, where the accuracy was roughly unchanged. Comparing between ToA and minimum amplitude ratios, it can be seen that ToA extracted using the S/L TA-AIC method gave lower error than using the signal amplitude ratios. When combined, both features gave lower localisation error than when used separately and were thus used for further comparison. 

### 6.3. Comparison Between Reference Method and Proposed Method

As the proposed method must be able to detect other impact cases besides soft impacts with acceptable accuracy, in this section, we compare the localisation accuracy between the benchmark method (ANNs with NSET ToA as input) and the proposed method (reference database with five sensor combinations with S/L TA-AIC ToA and minimum amplitude ratios as input) for all the cases collected in Section 2 (Table 5). As we know, the benchmark method is able to detect hard impacts (F1–F7 and C1–C4) with good accuracy [23], thus it would be desirable for the proposed method to have comparable accuracy with the benchmark method for these cases. Additionally, we looked at how the localisation error of soft impacts compared with hard impacts to see the effects of impactor stiffness on localisation accuracy.

To account for the cases with added noise (F7, F9, C4, and C6), high pass filtering (>700 Hz, Section 3.2) was conducted prior to ToA extraction. As the NSET method cannot reliably extract ToA for soft impacts (C5 and C6) when high pass filtering is conducted (Section 4.1), localisation for soft impacts was not conducted using the benchmark method (Table 5). 

**Figure 14:**
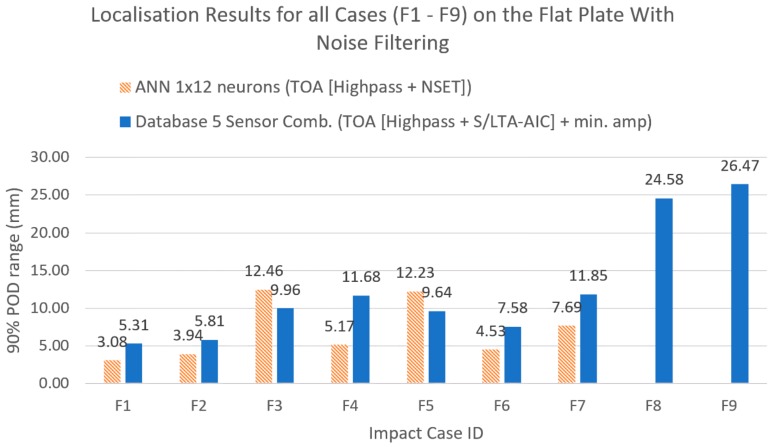
Comparison of localisation accuracy between the reference method (ANN with NSET extracted ToA input) and the proposed method (reference database with S/LTA-AIC extracted ToA and normalised minimum amplitude input) for the flat plate.

**Figure 15:**
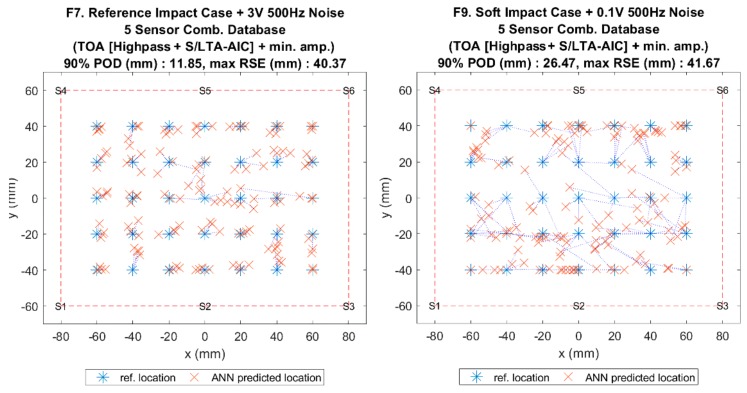
Visualisation of impact localisation for impacts with added noise for hard (F7, **left**) and soft (F9, **right**) impacts on the flat plate.

**Figure 16:**
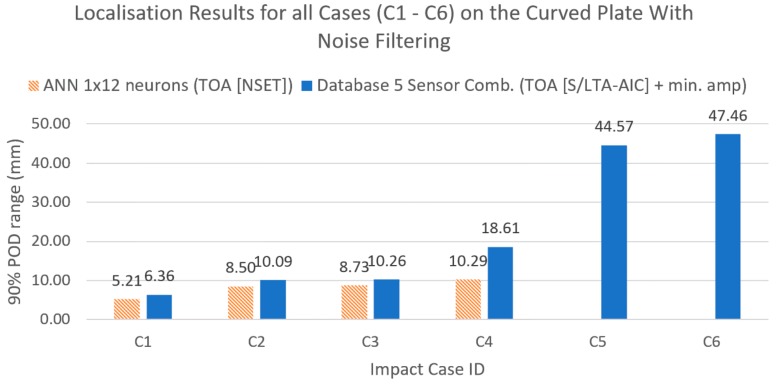
Comparison of localisation accuracy between the reference method (ANN with NSET extracted ToA input) and the proposed method (reference database with S/LTA-AIC extracted ToA and normalised minimum amplitude input) for the curved plate.

**Figure 17:**
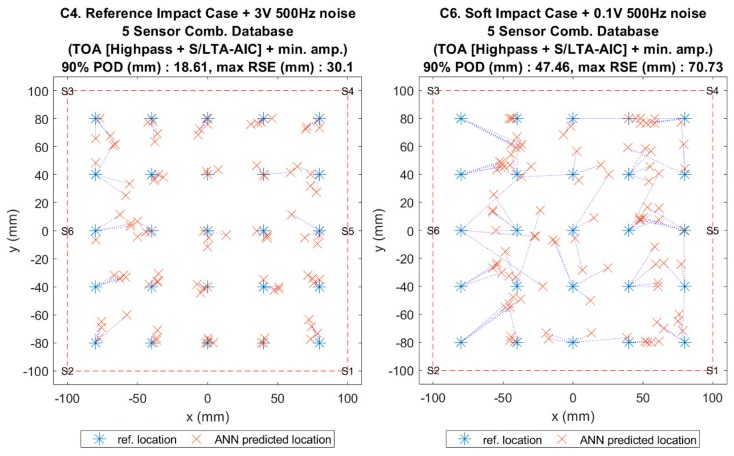
Visualisation of impact localisation for impacts with added noise for hard (C4, left) and soft (C6, right) impacts on the curved plate.

Figure 14, Figure 15, Figure 16 and Figure 17 show the results of the comparison between the benchmark and the proposed method for all impact cases collected. It can be seen that, for hard impacts, the error level was similar for the flat plate (Figure 14, F1–F7), whilst for the curved plate (Figure 16, C1–C4), the error level was also similar except for the case with added noise (C4), where the proposed method was less accurate. However, the error for these hard impacts was significantly lower than that of the soft impacts (F8, F9 and C5, C6) suggesting that variation caused by impactor stiffness was the limiting factor for localisation accuracy, as predicted earlier (Section 4 and Section 5). From Figure 10, Figure 14 and Figure 16, we can see that the proposed method was able to suppress the increase in error due to the variations from soft impacts to an acceptable level compared to the benchmark method. Additionally, it can be seen that there was little effect of added noise on soft impact localisation error (F9,C6), suggesting that the proposed method was able to accommodate for vibration noise.

To compare the localisation accuracy between plates with differing sensing area dimensions, we converted the 90% POD range into a circular detection area around the predicted impact location as a percentage of the whole sensing area [12,23]. For hard impacts, the maximum detection areas were 2.30% (F7) and 2.72% (C4) for the flat and the curved plates, respectively, indicating that the difference in configuration had no effect on localisation accuracy. For soft impacts, the maximum detection areas were 11.46% (F9) and 17.69% (C6) for the flat and the curved plates, respectively, suggesting that the difference in configuration in both plates influenced localisation accuracy. For these cases, the 90% POD ranges were 26.47 mm and 47.46 mm for the flat and the curved plates, which were similar to the experimental data point distances of the reference impact cases (20 mm and 40 mm for the flat and the curved plates, Figure 2) indicating that, for soft impacts, the accuracy was mostly dictated by the discretisation of the original data set of the reference database even though interpolation was applied. 

## 7. Conclusions

A parametric investigation of the effect of impactor stiffness as well as environmental and operational conditions on impact contact behaviour and the subsequently generated lamb waves in composite structures was conducted. From the tests carried out, it was observed that lower impactor stiffness generated a larger contact area (due to deformation) and subsequently generated a lamb wave with much lower amplitude and frequency due to said contact behaviour. 

These observations were utilised to develop a novel impact localisation method based on the reference database method and AIC ToA picker that is robust towards the variations due to differences in impactor stiffness. The proposed method was compared against a benchmark method based on ANNs and the NSET ToA extraction method, which is known to have good accuracy in locating impacts under various environmental and operational conditions but untested for different impactor stiffness. The results indicate that the proposed method had comparable accuracy (11.85–18.61 mm detection range) to the benchmark method (10.29–12.46 mm detection range) for hard impacts under various environmental and operational conditions when trained only using a single hard impact case. However, when tested with soft impacts, the benchmark method had very low accuracy (167.31–918.33 mm detection range) whilst the proposed method was able to maintain its accuracy at an acceptable level (26.18–45.48 mm detection range). It can be concluded that the proposed method is capable of detecting the location of impacts of varying stiffness and environmental and operational conditions using data from only a single impact case.

## Figures and Tables

**Table 1:** Impact cases for the flat plate.

Case	Case Name	Parameters^1^	Number of Samples	Energy (J)
F1	Reference Impact Case	Head = steel, h = 25, a = 90, m = 100, T = RT, n = none	140 (loc. 1-35 x 4 rep.)	0.025
F2	Increased Impact Height	Head = steel, h = 50, a = 90, m = 100, T = RT, n = none	140 (loc. 1-35 x 4 rep.)	0.050
F3	Angled Impact	Head = steel, h = 25, a = 45, m = 100, T = RT, n = none	140 (loc. 1-35 x 4 rep.)	0.025
F4	Increased Impact Mass	Head = steel, h = 25, a = 90, m = 200, T = RT, n = none	140 (loc. 1-35 x 4 rep.)	0.050
F5	Increased Impact Temperature	Head = steel, h = 25, a = 90, m = 100, T = 65, n = none	140 (loc. 1-35 x 4 rep.)	0.025
F6	Intermediate Impact Locations	Head = steel, h = 25, a = 90, m = 100, T = RT, n = none	48 (int. loc. 1-12 x 4 rep.)	0.025
F7	Reference Case + Artificial Noise	Head = steel, h = 25, a = 90, m = 100, T = RT, n = 500Hz 3V	140 (loc. 1-35 x 4 rep.)	0.025
F8	Soft Impact	Head = silicone, h = 50, a = 90, m = 100, T = RT, n = none	140 (loc. 1-35 x 4 rep.)	0.050
F9	Soft Impact + Artificial Noise	Head = silicone, h = 50, a = 90, m = 100, T = RT, n = 500Hz 0.1V	140 (loc. 1-35 x 4 rep.)	0.050

^1^ Notes: Head = impactor head material, h = vertical drop height (mm), a **=** impact angle (deg.), m = impactor mass (gr), T = temperature (°C, RT: room temperature, 22–24), n = noise (frequency [Hz], amplitude [V]).

**Table 2:** Impact cases for the curved plate.

Case	Case name	Parameters^1^	Number of Samples	Energy (J)
C1	Reference Impact Case	Head = steel, h = 25, a = 90, m = 100, T = RT, n = none	100 (loc. 1-25 x 3 rep.)	0.025
C2	Angled Impact	Head = steel, h = 25, a = 45, m = 100, T = RT, n = none	100 (loc. 1-25 x 4 rep.)	0.025
C3	Increased Impact Mass	Head = steel, h = 25, a = 90, m = 200, T = RT, n = none	100 (loc. 1-25 x 4 rep.)	0.050
C4	Reference Case + Artificial Noise	Head = steel, h = 25, a = 90, m = 100, T = RT, n = 500Hz 3V	100 (loc. 1-25 x 4 rep.)	0.025
C5	Soft Impact	Head = silicone, h = 50, a = 90, m = 100, T = RT, n = none	100 (loc. 1-25 x 4 rep.)	0.050
C6	Soft Impact + Artificial Noise	Head = silicone, h = 50, a = 90, M = 100, T = RT, n = 500Hz 0.1V	100 (loc. 1-25 x 4 rep.)	0.050

^1^ Notes: Head = impactor head material, h = vertical drop height (mm), a = impact angle (deg.), m = impactor mass (gr), T = temperature (°C, RT: room temperature, 22–24), n = noise (frequency [Hz], amplitude [V]).

**Table 3:** Test cases for comparison between localization methods.

Test Impact Case	Noise Filtering	Feature Used	Localisation Method	Training/Reference Database^1^
Soft impact (F8 and C5)	No	ToA (NSET)	ANN, 1 × 12 hidden layer	F1 and C1
Soft impact (F8 and C5)	No	ToA (NSET)	Database, 6 sensors	F1 and C1
Soft impact (F8 and C5)	No	ToA (NSET)	Database, 5 sensor comb.	F1 and C1
Soft impact (F8 and C5)	No	ToA (NSET)	Database, 4 sensor comb.	F1 and C1
Soft impact (F8 and C5)	No	ToA (NSET)	Database, 3 sensor comb.	F1 and C1

^1^ Note: F1 for flat plate impacts and C1 for curved plate impacts.

**Table 4:** Test cases for comparison between input features for localisation.

Test Impact Case	Noise Filtering	Feature Used	Localisation Method	Training/Reference Database^1^
Soft impact (F8 and C5)	No	ToA (NSET)	ANN, 1x12 hidden layer	F1 and C1
Soft impact (F8 and C5)	No	ToA (NSET)	Database, 5 sensor comb.	F1 and C1
Soft impact (F8 and C5)	No	ToA (S/L TA - AIC)	Database, 5 sensor comb.	F1 and C1
Soft impact (F8 and C5)	No	Min. amp. ratio	Database, 5 sensor comb.	F1 and C1
Soft impact (F8 and C5)	No	ToA (S/L TA - AIC) + Min. amp. ratio	Database, 5 sensor comb.	F1 and C1

^1^ note: F1 for flat plate impacts and C1 for curved plate impacts. ANN = artificial neural network.

**Table 5:** Test cases for comparison between the reference and the proposed method.

Test Impact Case	Case ID	Noise Filtering	Reference Method ^a^	Proposed Method ^b^	Training/ Reference Database ^c^
Reference Impact Case	F1 and C1	>700 Hz	Yes	Yes	F1 and C1
Increased Impact Height	F2	>700 Hz	Yes	Yes	F1 and C1
Angled Impact	F3 and C2	>700 Hz	Yes	Yes	F1 and C1
Increased Impact Mass	F4 and C3	>700 Hz	Yes	Yes	F1 and C1
Increased Impact Temperature	F5	>700 Hz	Yes	Yes	F1 and C1
Intermediate Impact Locations	F6	>700 Hz	Yes	Yes	F1 and C1
Reference Case + Artificial Noise	F7 and C4	>700 Hz	Yes	Yes	F1 and C1
Soft Impact	F8 and C5	>700 Hz	-	Yes	F1 and C1
Soft Impact + Artificial Noise	F9 and C6	>700 Hz	-	Yes	F1 and C1

Note: a. Reference method: ANN (1 x 12 hidden layer) with ToA (NSET) input; b. Proposed method: database (five sensor combination) with ToA (S/L TA-AIC) and minimum amplitude ratio.; **c.** F1 for flat plate impacts and C1 for curved plate impacts.

## References

[1] Kim H., DeFrancisci G., Chen Z.M., Rhymer J. Impact damage formation on composite aircraft structures. Proceedings of the UCSD FAA JAMS Paper, Technical Review Meeting.

[2] Fawcett A.J., Oakes G.D. Boeing composite airframe damage tolerance and service experience. Proceedings of the Composite Damage Tolerance and Maintenance Workshop.

[3] Erchart D., Ostrom L.T., Wilhelmsen C.A. (2004). Visual detectibility of dents on a composite aircraft inspection specimen: an initial study. Int. J. Appl. Aviat. Stud..

[4] Abrate S. (2005). Impact On Composite Structures.

[5] Fu H., Seno A.H., Khodaei Z.S., Ferri Aliabadi M.H. Design of a Wireless Passive Sensing System for Impact Detection of Aerospace Composite Structures. Proceedings of the 2018 5th IEEE International Workshop on Metrology for AeroSpace (MetroAeroSpace).

[6] Bekas D., Sharif-Khodaei Z., Aliabadi M.H. (2018). An Innovative Diagnostic Film for Structural Health Monitoring of Metallic and Composite Structures. Sensors.

[7] Yuan S., Ren Y., Qiu L., Mei H. (2016). A Multi-Response-Based Wireless Impact Monitoring Network for Aircraft Composite Structures. IEEE Trans. Ind. Electron..

[8] Coverley P.T., Staszewski W.J. (2003). Impact damage location in composite structures using optimized sensor triangulation procedure. Smart Mater. Struct..

[9] De Simone M.E., Ciampa F., Boccardi S., Meo M. (2017). Impact source localisation in aerospace composite structures. Smart Mater. Struct..

[10] Beligni A., Sbarufatti C., Gilioli A., Cadini F., Giglio M. (2019). Robust Identification of Strain Waves due to Low-Velocity Impact with Different Impactor Stiffness. Sensors.

[11] Yue N., Sharif Khodaei Z. (2016). Assessment of Impact Detection Techniques for Aeronautical Application: ANN vs. LSSVM. J. Multiscale Model..

[12] Sharif Khodaei Z., Ghajari M., Aliabadi M.H. (2012). Determination of impact location on composite stiffened panels. Smart Mater. Struct..

[13] Jang B.-W., Kim C.-G. (2016). Impact localization on a composite stiffened panel using reference signals with efficient training process. Compos. Part B Eng..

[14] Li H., Wang Z., Forrest J.Y.-L., Jiang W. (2018). Low-Velocity Impact Localization on Composites Under Sensor Damage by Interpolation Reference Database and Fuzzy Evidence Theory. IEEE Access.

[15] Park J., Ha S., Chang F.-K. (2009). Monitoring Impact Events Using a System-Identification Method. AIAA J..

[16] Shrestha P., Park Y., Kwon H., Kim C.-G. (2017). Error outlier with weighted Median Absolute Deviation threshold algorithm and FBG sensor based impact localization on composite wing structure. Compos. Struct..

[17] Sh Sharif Khodaei Z., Ferri Aliabadi M.H. (2018). Impact Detection and Identification with Piezoceramic Sensors: Passive Sensing. Computational and Experimental Methods in Structures.

[18] Kundu T., Das S., Jata K.V., Kundu T. (2007). An improved technique for locating the point of impact from the acoustic emission data. Health Monitoring of Structural and Biological Systems 2007.

[19] Kundu T., Nakatani H., Takeda N. (2012). Acoustic source localization in anisotropic plates. Ultrasonics.

[20] Zhu J., Parvasi S.M., Ho S.C.M., Patil D., Ge M., Li H., Song G. (2017). An innovative method for automatic determination of time of arrival for Lamb waves excited by impact events. Smart Mater. Struct..

[21] Al-Jumaili S.K., Pearson M.R., Holford K.M., Eaton M.J., Pullin R. (2016). Acoustic emission source location in complex structures using full automatic delta T mapping technique. Mech. Syst. Sig. Process..

[22] Ciampa F., Meo M. (2012). Impact detection in anisotropic materials using a time reversal approach. Struct. Health Monit..

[23] Seno A.H., Sharif Khodaei Z., Aliabadi M.H.F. (2019). Passive sensing method for impact localisation in composite plates under simulated environmental and operational conditions. Mech. Syst. Sig. Process..

[24] Mallardo V., Aliabadi M.H., Khodaei Z.S. (2013). Optimal sensor positioning for impact localization in smart composite panels. J. Intell. Mater. Syst. Struct..

[25] Sanchez N., Meruane V., Ortiz-Bernardin A. (2016). A novel impact identification algorithm based on a linear approximation with maximum entropy. Smart Mater. Struct..

[26] Salmanpour M.S., Sharif Khodaei Z., Aliabadi M.H.F. (2017). Impact Damage Localisation with Piezoelectric Sensors under Operational and Environmental Conditions. Sensors.

[27] Croxford A.J., Moll J., Wilcox P.D., Michaels J.E. (2010). Efficient temperature compensation strategies for guided wave structural health monitoring. Ultrasonics.

[28] Ha S., Lonkar K., Mittal A., Chang F.-K. (2010). Adhesive Layer Effects on PZT-induced Lamb Waves at Elevated Temperatures. Struct. Health Monit. Int. J..

[29] Sung D.-U., Oh J.-H., Kim C.-G., Hong C.-S. (2000). Impact Monitoring of Smart Composite Laminates Using Neural Network and Wavelet Analysis. J. Intell. Mater. Syst. Struct..

[30] Kim I.-G., Lee H.-Y., Kim J.-W. (2005). Impact Damage Detection in Composite Laminates Using PVDF and PZT Sensor Signals. J. Intell. Mater. Syst. Struct..

[31] Caprino G., Lopresto V., Leone C., Papa I. (2011). Acoustic emission source location in unidirectional carbon-fiber-reinforced plastic plates with virtually trained artificial neural networks. J. Appl. Polym. Sci..

[32] Jang B.-W., Kim C.-G. (2019). Impact localization of composite stiffened panel with triangulation method using normalised magnitudes of fiber optic sensor signals. Compos. Struct..

[33] Maeda N. (1985). A method for reading and checking phase times in autoprocessing system of seismic wave data. Zisin.

[34] Stevenson P.R. (1976). Microearthquakes At Flathead Lake, Montana: A Study Using Automatic Earthquake Processing. Bull. Seismol. Soc. Am..

[35] Allen R.V. (1982). Automatic Phase Pickers: Their Present Use and Future Prospects. Bull. Seismol. Soc. Am..

[36] Shang X., Li X., Morales-Esteban A., Dong L. (2018). An Improved P-Phase Arrival Picking Method S/L-K-A with an Application to the Yongshaba Mine in China. Pure Appl. Geophys..

[37] Hossain M.S., Ong Z.C., Ismail Z., Noroozi S., Khoo S.Y. (2017). Artificial neural networks for vibration based inverse parametric identifications: A review. Appl. Soft Comput..

[38] Jang B.-W., Lee Y.-G., Kim J.-H., Kim Y.-Y., Kim C.-G. (2012). Real-time impact identification algorithm for composite structures using fiber Bragg grating sensors. Struct. Control Hlth..

[39] Kim J.-H., Kim Y.-Y., Park Y., Kim C.-G. (2015). Low-velocity impact localization in a stiffened composite panel using a normalised cross-correlation method. Smart Mater. Struct..

