# Correction: Seno, A.H.; Ferri Aliabadi, M.H. Impact Localisation in Composite Plates of Different Stiffness Impactors under Simulated Environmental and Operational Conditions. *Sensors* 2019, *19*, 3659

**DOI:** 10.3390/s20226410

**Published:** 2020-11-10

**Authors:** Aldyandra Hami Seno, M.H. Ferri Aliabadi

**Affiliations:** Department of Aeronautics, Imperial College London, Exhibition Road, South Kensington, London SW7 2AZ, UK; a.hami-seno16@imperial.ac.uk

The author wishes to make the following correction to this paper [1] due to mislabeling (as an artifact from our code) and would like to replace the following figures:

**Figure 11:**
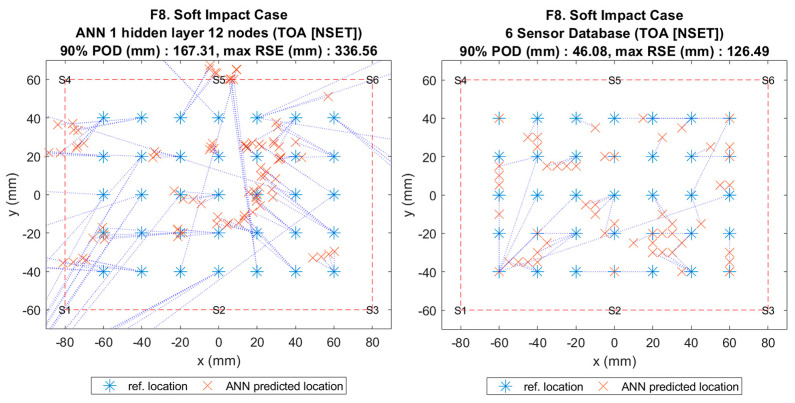
Visualisation of impact localisation for soft impacts (F8) using different methods: ANN (left) and reference database (right).

**Figure 13:**
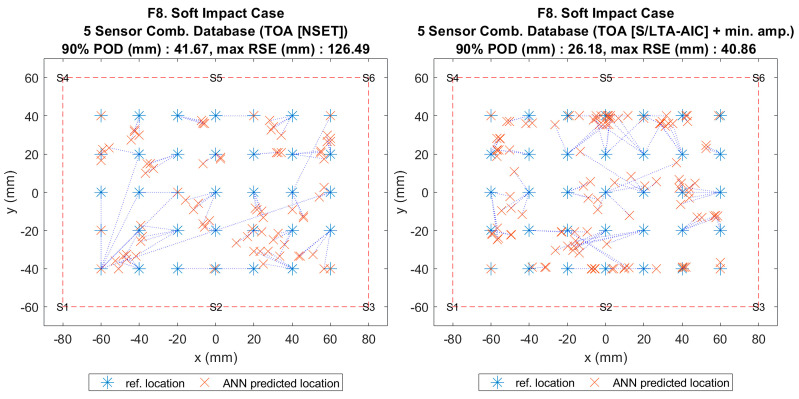
Visualisation of impact localisation for soft impacts (F8) using different input features: NSET extracted ToA (left) and S/LTA-AIC extracted ToA with normalized minimum amplitude (right).

**Figure 15:**
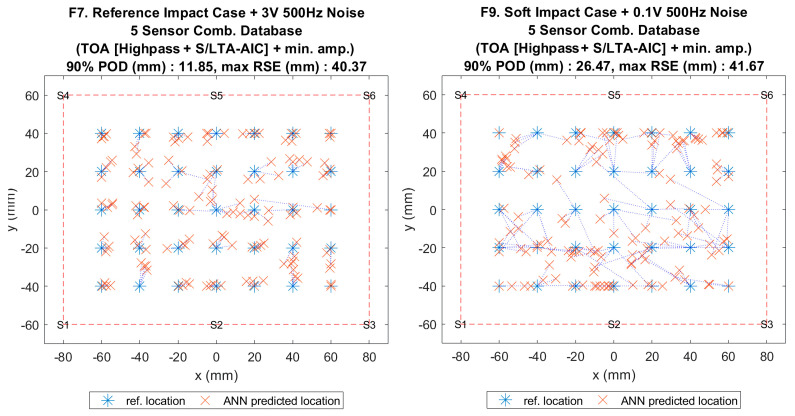
Visualisation of impact localisation for impacts with added noise for hard (F7, left) and soft (F9, right) impacts on the flat plate.

**Figure 17:**
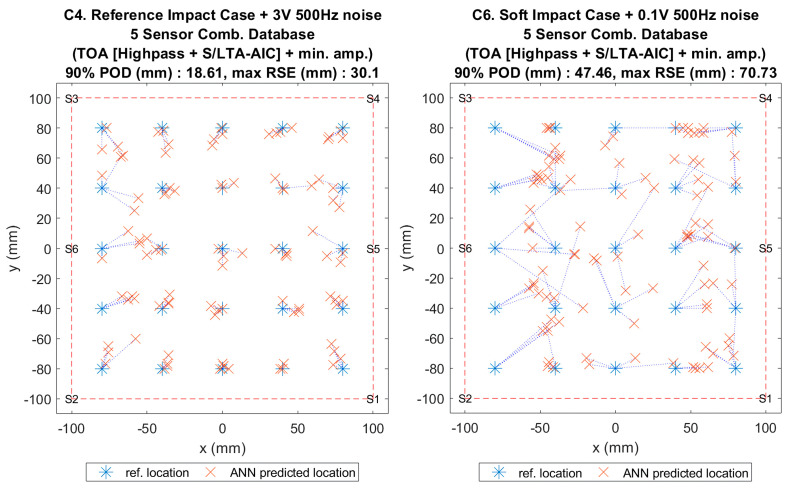
Visualisation of impact localisation for impacts with added noise for hard (C4, left) and soft (C6, right) impacts on the curved plate.

With the following corrected Figure 11, 13, 15 and 17:

The reason being that the localization results shown in these figures are not only from ANNs but also from the proposed database (DTB) method and as such it is more appropriate that the label should be “predicted locations” rather than “ANN predicted locations”. This change will not affect any results or findings in the paper.

We apologize for any convenience caused by this change.

## Figures and Tables

**Figure 11:**
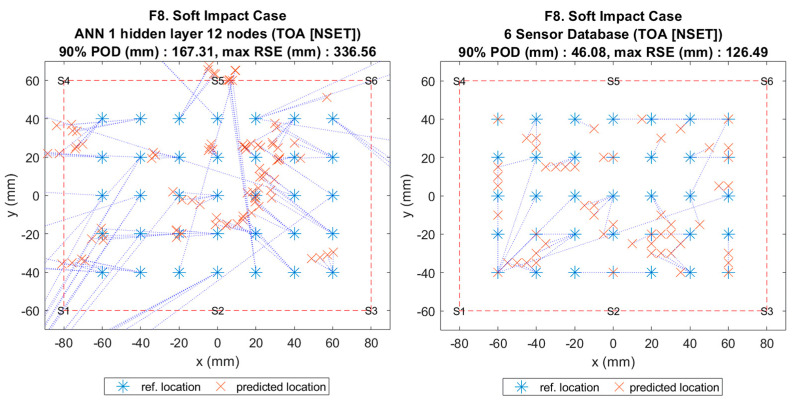
Visualisation of impact localisation for soft impacts (F8) using different methods: ANN (left) and reference database (right).

**Figure 13:**
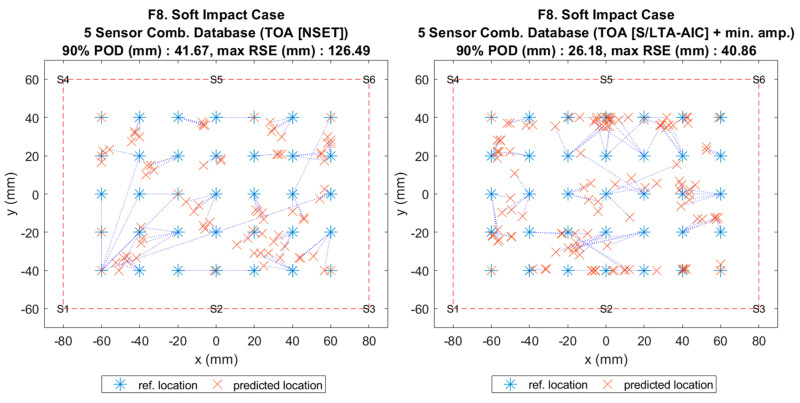
Visualisation of impact localisation for soft impacts (F8) using different input features: NSET extracted ToA (left) and S/LTA-AIC extracted ToA with normalized minimum amplitude (right).

**Figure 15:**
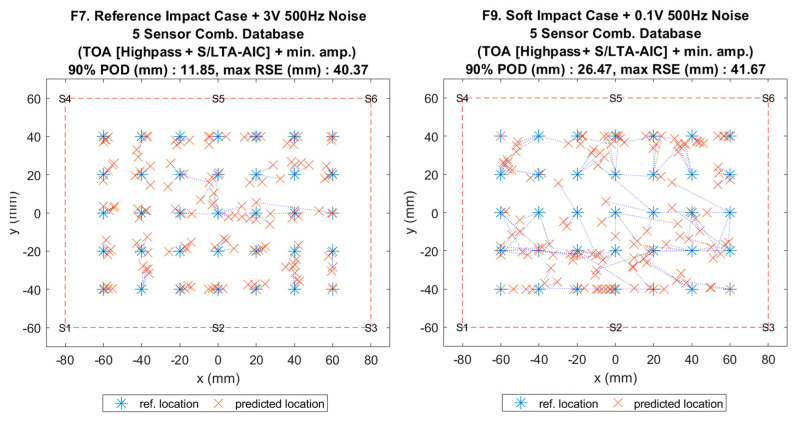
Visualisation of impact localisation for impacts with added noise for hard (F7, left) and soft (F9, right) impacts on the flat plate.

**Figure 17:**
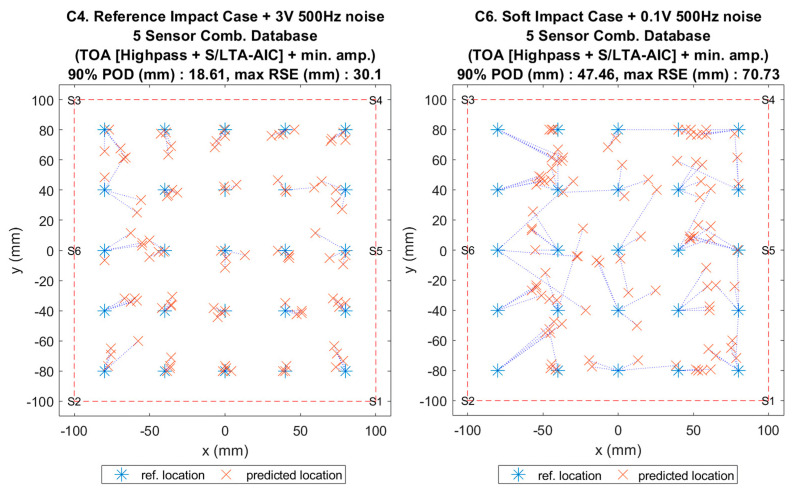
Visualisation of impact localisation for impacts with added noise for hard (C4, left) and soft (C6, right) impacts on the curved plate.
